# The role of SEAD project intervention in viral suppression of HIV/AIDS patients with follow-up and adherence barriers

**DOI:** 10.7448/IAS.15.6.18093

**Published:** 2012-11-11

**Authors:** L Elías Casado, M Pérez Elías, D López Pérez, M Pumares Álvarez, M Martinez-Colubi, A Moreno Zamora, A Muriel, F Dronda, P Marti-Belda, C Gómez-Ayerbe, M Rodriguez Sagrado, S Moreno Guillén

**Affiliations:** 1 Hospital Ramón y Cajal, IRYCIS, Infectious Diseases Department, Madrid, Spain; 2 Hospital Ramón y Cajal, IRYCIS, Department of Biostatistics, Madrid, Spain; 3 Hospital Ramón y Cajal, IRYCIS, Pharmacy, Madrid, Spain

## Abstract

**Purpose of study:**

Irregular FUP/ADH were associated with virologic failure [1] leading to an increase in mortality [2]. SEAD was a multidimensional intervention project, designed from the patient's perspective, to specifically attend patients with poor FUP/ ADH in an HIV/AIDS outpatient clinic.

**Methods:**

From Jan 2006 to May 2010, patients with poor FUP/ADH were offered SEAD inclusion, all were evaluated by a nurse or a psychologist (adherence collaborators) who assessed all the reasons and barriers precluding a correct FUP/ADH. For each identified problem, different interventions were planned, using our own resources or coordinating others. Follow-up was censored in Nov 2011. Univariate and multivariable models were performed to evaluate the influence of SEAD intervention in virological suppression (HIV-ARN <1.7 log copies/mL) at the end of follow-up.

**Summary of results:**

Overall, 242 patients were assessed: mean age 46 years, 78% men, 69% IDU, 51% AIDS, baseline ADH >90% 29.3%; median CD4 cell count 333 [164–536] cells/mL and HIV-RNA <1.57 45%. Patients were admitted in SEAD due to poor ADH 15%, FUP problems 21%, both FUP/ADH 53% and to prevent poor ADH or FUP 11%. Main reasons driving poor FUP/ADH were severe biopsychosocial problems 26%, severe drug and/or alcohol abuse 23%, logistic problems 21.3%, other psychiatric disorders 14%, oversights 10%, unknown 3% and antiretroviral intolerance 2%. Cocaine/heroin and alcohol abuse was reported by 33% and 16%. Only 57% of patients received >50% of planned interventions. After a median follow-up of 3.9 (3.27–4.43) years 218 patients received 8 (3–12) interventions/year, 95% evaluation interview and 30% psychological counselling (3 sessions/year [2–5]). Virological suppression was achieved by 67% of patients. In logistic regression analysis an intervention higher than 50% of planned HR 0.220 [IC 95% (0.112–0.44)] and receiving psychological counselling HR 0.44 [IC 95% (0.20–0.97)] were independent predictors of virological suppression whereas alcohol 3.11 (95% CI 1.24–7.80) and severe biopsychosocial problems HR 2.39 (95% CI 1.134–5.040) were associated with worse virological response, after adjusting for age, alcohol or cocaine abuse, degree of adherence, baseline virological suppression, median follow–up, intravenous acquisition of HIV, and family support.

**Conclusions:**

General and psychological SEAD intervention resulted in higher virological suppression in patients with severe follow-up and adherence barriers.
